# A biomimetic synthetic nanofiber-based model for anterior cruciate ligament regeneration

**DOI:** 10.3389/fbioe.2022.969282

**Published:** 2022-10-26

**Authors:** Abass Ojo Adeoye, Fariza Mukasheva, Smail Smatov, Bakhytbol Khumyrzakh, Sanazar Kadyr, Zarina Shulgau, Cevat Erisken

**Affiliations:** ^1^ Department of Chemical and Materials Engineering, Nazarbayev University, Astana, Kazakhstan; ^2^ National Center for Biotechnology, Laboratory of Toxicology and Pharmacology, Astana, Kazakhstan

**Keywords:** anterior cruciate ligament, collagen fibrils, rat, electrospinning, PCL, nanofiber, bimodal diameter distribution, tissue regeneration

## Abstract

Reconstructed ACL cannot completely restore its functions due to absence of physiologically viable environment for optimal biomaterial-cell interaction. Currently available procedures only mechanically attach grafts to bone without any biological integration. How the ACL cells perform this biological attachment is not fully understood partly due to the absence of appropriate environment to test cell behavior both *in vitro* and *in vivo*. Availability of biomimetic models would enable the scientists to better explore the behavior of cells at health and during tissue healing. In this study, it is hypothesized that the collagen fibril diameter distribution in rat ACL changes from a bimodal distribution in the healthy ACL to a unimodal distribution after injury, and that this change can be mimicked in synthetic nanofiber-based constructs. This hypothesis was tested by first creating an injured rat ACL model by applying a mechanical tensile force to the healthy ACL tissue until rupture. Secondly, the collagen fibril diameter distributions of healthy and injured ACL tissue were determined, and polycaprolactone (PCL) constructs were created to mimic the distributions of collagen fibrils in healthy and injured tissues. Findings reveal that the fiber diameter distribution of aligned bimodal PCL constructs were similar to that of the collagen fibrils in native ACL tissue. This study is significant because suggested bimodal and unimodal fibrous model constructs, respectively, represent a healthy and injured tissue environment and the behavior of ACL cells cultured on these constructs may provide significant input on ACL regeneration mechanism.

## 1 Introduction

The Anterior Cruciate Ligament (ACL) in the knee joint, which acts as a resistor and stabilizer for anterior tibial and rotational stresses, is essential for knee joint stability and kinematics, and is one of the most frequently injured components in the knee joint (Duthon et al., 2006). The ACL is a hypocellular and weakly vascularized connective tissue that is mostly made up of aligned type 1 collagen fibrils and surrounded by fibroblasts, elastin, and glycoproteins as well as glycosaminoglycans. When the knee is stretched, the posterolateral bundle will get tight and the anteromedial bundle will relax depending on the viscoelastic and biomechanical properties of the ACL, which are controlled mainly by the collagen fibrils (Petersen and Zantop, 2007). The ACL is positioned diagonally at the center of the knee and a tear or rupture of the collagen fibrils of the ACL results in structural and morphological changes mainly in the form of a variation in the diameter distribution of collagen fibrils and a reduction in the mean diameter (Ochi et al., 1999; Beisbayeva et al., 2021). This can lead to a decrease in mechanical resistance to force, and instability in the joint, which can, then, be followed by tears to the meniscus as well as initiation of osteoarthritis (Zantop et al., 2006).

In this perspective, collagen diameter is an important ACL feature since it ultimately determines the tissue’s tensile strength and stability (Zhu et al., 2012). According to prior research examining the relationship between mechanical properties and fibril diameter (Marieswaran et al., 2018), a physiological range of fibril diameter seems essential for optimal ACL function. Under tensile loading, the stress-strain behavior of ACL exhibits a tri-phasic pattern that includes the toe, linear, and yield zones (Allen et al., 1999). The pattern in the fibrils in the toe region straightens out at low stresses, requiring lower pressures. In the linear area, at the beginning of elastic deformation, the resistance pressure steadily increases. The yield area signifies the beginning of irreversible deformation, and due to the tearing of collagen fibrils at this point, stress diminishes, resulting in the rupture of the ligament (Allen et al., 1999).

Previous investigations on the collagen fibril diameter of healthy ACL tissue clearly demonstrate the presence of two peaks in the healthy human (Zhu et al., 2012), rabbit (Hart et al., 1999) and bovine ACL (Beisbayeva et al., 2021) tissues. However, the distribution of the collagen fibril diameter in health and after injury has not been reported for these species except for bovine that was investigated by our group. The collagen fibril diameter distribution of healthy bovine ACL demonstrated peaks at 73.3 ± 11.5 nm and 213 ± 11.5 nm which then disappeared, upon injury, to form a single peak at 100 ± 20 nm, with a decrease in the mean diameter (Beisbayeva et al., 2021). For a broader aim at generating a generalizable behavior for the ACL fibril diameter distribution of other species in health and injury, this study reports the characterization of rat ACL collagen fibril diameter distribution before and after injury. It is hypothesized that the collagen fibril diameter distribution of rat ACL tissue has a bimodal distribution in health and changes to unimodal upon injury. Rat ACLs, like human ACLs, provide stability during joint movement. Due to anatomical similarities between rats and human (Ma et al., 2015), this study employed rats as animal models to investigate the effect of injury on the diameter of collagen fibrils in the ACL tissue.

Injuries due to ACL rupture are reported to occur more than 200,000 times every year in the United States (Mall et al., 2014). Because of its hypocellular and minimally vascularized nature, and the frequency of motion, ACL injuries do not regenerate, and their healing process results in the development of a scar tissue. The ACL is frequently injured in sports as a result of axial loading coupled with the rotation of the valgus-internal angulation of the knee (Lu and Thomopoulos, 2013), and currently available medical treatments, i.e., reconstruction procedures, involving grafts of different sources are unable to effectively heal the ACL injuries (Samuelsson et al., 2009). The autologous grafts seem to offer the best possible service given their structural, morphological and biological properties, however, they only come at the expense of morbidity and harvesting a piece of tissue from the patient. Allografts and xenografts, on the other hand, carry a higher risk in terms of disease transmission. Thus, synthetic grafts remain as a plausible option due to their tailorable structural and morphological properties, when combined with their enhanced biological compatibility. However, the current state-of-the-art related to synthetic grafts seems to be missing an important physiological characteristics of the native ACL tissue, i.e., the organization and diameter distribution of collagen fibrils. This study, therefore, aims at creating a model construct to, later, investigate ligament cell response and better understand ACL regeneration mechanism.

Given the nanometer scale of the collagen fibrils in ACL (Neurath and Stofft, 1992; Beisbayeva et al., 2021), nanofiber-based synthetic grafts can add biomimicry to the regenerative engineering efforts of ACL reconstruction because of their architectural similarities to the extracellular matrix, as well as their adaptable chemical and physical properties for influencing cell response and behavior (Ashammakhi et al., 2012; Han et al., 2021).

The electrospinning technology has been proven to be capable of creating fibers on the nanometer and micrometer scale (Erisken et al., 2013; Han et al., 2021), which represent the diameter scale of collagen fibrils and fibers, respectively. However, the challenge with the electrospinning is the creation of controlled ranges in both nanofiber and microfiber scales to mimic not only the diameter range but also the distribution of diameters in the desired range. In our previous efforts, we investigated the possibility of mimicking the range of collagen fibrils seen in bovine ACL tissue using polycaprolactone (PCL) as the material of construction of the synthetic models for ACL regeneration (Beisbayeva et al., 2021). However, replicating the distribution of ACL collagen fibrils smaller than 100 nm has not been possible due to limitations related to the material properties in the process of electrospinning. With continued efforts on the formulation, fabrication of models from PCL nanofibers covering the sub-100 nm range has now been realized (Mukasheva, 2019). Different biomaterials including poly (lactic-co-glycolic acid), poly (lactic acid) and polycaprolactone have been previously utilized as scaffolding material for ACL tissue engineering (Alshomer et al., 2018; Sensini and Cristofolini, 2018). Due to its presence on the list of materials approved by the Food and Drug Administration, USA, to be used as materials of construction for a variety of biomedical devices, ease of electrospinability (Erisken et al., 2008a), affordable cost, sufficiently long degradation time, and suitability in terms of mechanical properties (Gurlek et al., 2017), PCL was selected as the material of synthetic nanofiber-based model in this study. With the aim to fabricate a nanofiber-based synthetic model, it is hypothesized that fiber diameter distribution of this model will be similar to that of healthy ACL tissue. If this hypothesis comes true, for the first time, a synthetic construct will appear in the biomaterials field to both qualitatively and quantitatively mimic the structural and morphological properties of native ACL tissue to be used as a model to study the cell response.

In this work, ACL injuries were created using an *ex vivo* extensional deformation on rat knee joints, and the collagen fibril diameter distribution and mean diameter were determined for both injured and healthy ACL tissues using TEM images. The PCL model constructs were fabricated using electrospinning technique and evaluated for both diameter distribution and mean fiber diameter. Biomechanical properties of the healthy ACL tissues were characterized and compared with those of PCL model constructs.

## 2 Materials and methods

This research study involves three major experimental studies: 1) harvesting ACL tissues from rats, 2) fabricating electrospun nanofiber constructs, and 3) their characterization in terms of diameter of structural components using TEM/SEM, and tensile biomechanical properties.

### 2.1 Materials

All chemicals were acquired from Sigma Aldrich, and their product numbers are listed below. Glutaraldehyde (Sigma Aldrich, #G5882), osmium tetroxide (1%, Sigma Aldrich, #75633), Phosphate buffer solution (Sigma Aldrich, #P5244), ethanol (Sigma Aldrich, #E7023), propylene oxide (Sigma Aldrich, #82320), epoxy embedding medium 812 substitute (Sigma Aldrich, #45345), polycaprolactone (Sigma-Aldrich, #440744), dichloromethane (Sigma Aldrich, #270997), N-N-Dimethylformamide (Sigma Aldrich, #319937), epoxy embedding medium hardener DDSA (Sigma Aldrich, #45346), epoxy embedding medium hardener MNA (Sigma Aldrich, #45347), epoxy embedding medium accelerator DMP 30 (Sigma Aldrich, #45348), Polycaprolactone (Sigma-Aldrich, ##440744), acetic acid (#270725), formic acid (90%, #110854), pyridine (Sigma-Aldrich #270970).

### 2.2 Harvesting the ACL tissue

**FIGURE 1:**
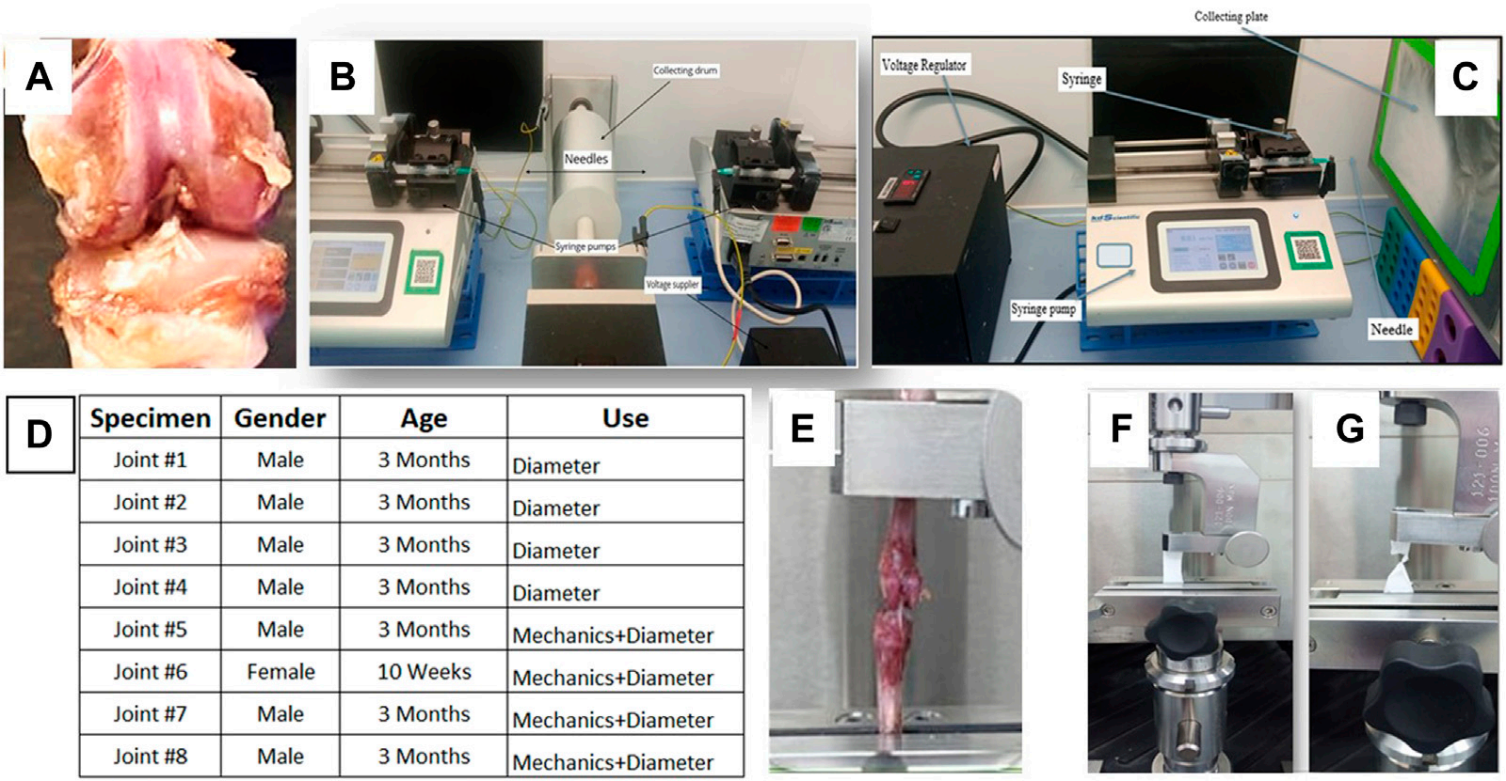
**(A)** Gross view of the rat knee joint, electrospinning set-up for **(B)** aligned bimodal and **(C)** random unimodal PCL constructs, **(D)** gender and age of rat ACL tissues, mechanical testing apparatus for **(E)** rat ACL and **(F,G)** PCL constructs.

The rat limbs were obtained from the National Biotechnology Center (Astana, Kazakhstan) shortly after the animals were slaughtered, and preserved at −20°C until needed. Before testing, the frozen knee joints were thawed at room temperature. A surgical blade was used to remove all tendons and ligaments from the joint except the ACL (Figure 1A).

### 2.3 Tensile tests for ACL and creating ACL injury

A uniaxial material testing machine with a 1 kN load cell (MTS Criterion Model 43, MTS Systems Co., Eden Prairie, MN, United States) was used to apply tensile deformation to generate biomechanical parameters of ACL tissues. A custom-made jaw assembly was used to mount the Tibia-ACL-Femur joint to the unit, and the ACL’s axis was aligned with the load axis of the testing equipment (Figure 1E). An initial load of 0.3N was applied to straighten the specimens and the specimens were stretched till failure at a continuous crosshead speed of 5 mm/min. Biomechanical characteristics were tested on a total of four rat joints (Figure 1D, *n* = 4). Upon reaching the state of failure, the knee joints were removed, and the ruptured ACL was used to represent the injured ACL tissue, and all four specimens were employed in fibril diameter measurements to evaluate fibril diameter distribution of injured ACL tissue. This *ex-vivo* ACL injury model was previously applied successfully (Beisbayeva et al., 2021).

### 2.4 Preparing rat ACL for TEM characterization

The diameter distributions of collagen fibrils were measured using Transmission Electron Microscopy (TEM) images of tissue sections. First, the ACL tissue was located, it was stretched for better visibility, and it was removed from the joint to provide a specimen with dimensions of approximately 2 mm × 2 mm × 2 mm from the middle section of the tissue (*n* = 4). To prepare injured ACL tissue, after rupture of the ACL tissues, a representative specimen with the size of 2 mm× 2 mm × 2 mm was harvested from the injured ACL tissues from the region close to the point of rupture (*n* = 4).

To prepare the TEM specimens, a method reported elsewhere was employed (Beisbayeva et al., 2021). Briefly, first, the specimens were preserved with a 2.5 percent glutaraldehyde solution to prevent any possible modification in the joint structure during processing. The specimens were then held at ambient temperature for fixation and then gradually cooled down to 4°C. To slow down autolytic processes and minimize tissue shrinking, this form of cooling is required. Phosphate buffer solution (PBS) was used to wash the specimens three times for 10 minutes each time. A subsequent fixation with 1 percent osmium tetroxide for 2 h was used to give the specimen additional stability. Osmium tetroxide was employed as both a fixative and a contrasting substance. Specimens were washed twice in PBS for 10 min each time after fixation. As a transitional solvent, the specimens were dehydrated using a graded series of 50 percent ethanol for 40 min, 70 percent ethanol for 12 h, 96 percent ethanol for 20 min twice, 100 percent ethanol for 15 min twice, and a mixture of 100 percent ethanol and propylene oxide for 10 min. To ensure a seamless transition, a graded ethanol sequence was used to prevent any alterations in tissue structure. After that, different resin and propylene oxide combinations were used to penetrate the dried specimens. Various amounts of epoxy mix medium components 812, DDSA (Dodecenylsuccinic anhydride) and MNA (Methyl nadic anhydride) were used to make the resin. Infiltration was used to fill blocks of samples with resin to make them hard enough to bear pressure during sectioning and cutting. Samples were submerged in a 1:1 mixture of resin and propylene oxide for 2 h at 37°C. After 2 h at 37°C, the mixture was adjusted to a 3:1 ratio, followed by 12 h of pure resin. Different resin and propylene oxide mixes were submerged in molds for 24 h in the next step. The implanted samples were polymerized over the course of 2 days at 60°C.

Finally, using an ultra-microtome (Boeckeler PT-PC PowerTome Ultramicrotome, United States), a thin slice perpendicular to the longitudinal axis of the ligament was cut and utilized to complete the experiment. By pressing the block to the sides in regulated increments over a diamond knife, this one-of-a-kind piece of equipment may be used to cut specimen components. The chopped portions are collected and filled with distilled water on this piece of equipment. Each slice was selected to be roughly 60 nm in thickness. A Transmission Electron Microscope (JEOL JEM-1400 Plus 120 kV TEM) was utilized to capture high magnification pictures of ACL sections. Perpendicular cuts of fibrils were optimized to obtain cross-sectional pictures representing specimens from various regions of the collected tissue.

### 2.5 Measurement of ACL fibril diameter and organization

On the TEM images, 10 equally separated parallel lines were created, and the diameter as well as aspect ratio (short axis normalized by the long axis) of fibrils crossing these lines were measured. The diameter and aspect ratio of each fibril were measured using the ImageJ image processing program (National Institutes of Health, US). The fibrils in each portion were counted to a minimum of 100 per section, with three sections utilized for each joint. Over 300 typical readings were acquired for each of the two groups of ACL tissues, both in healthy and in injured condition. Finally, for each group, the diameter distribution, mean diameter, and ranges were calculated using the data (for healthy and injured ACL). The aspect ratio measured here indirectly evaluates the alignment of the collagen fibrils. An aspect ratio of 1 defines perfect alignment with a perfect circle of collagen fibril cross-section, and the smaller the value of aspect ratio, the higher the degree of deviation from the perfect alignment. SEM images of PCL constructs were similarly used after fabrication to calculate the diameter of the fibers in the constructs.

### 2.6 Nanofiber model construct fabrication

The PCL constructs were fabricated using polycaprolactone with a molecular weight of 80,000 g/mol. PCL solutions in different concentrations were produced in preparation for the electrospinning process. Bimodal and unimodal distributions were obtained by dissolving PCL in an acetic acid and formic acid solution with pyridine to get a final volume of 1 ml (Table 1). The solutions were prepared by mixing the materials at 40°C for 2 h while stirring constantly at 1,500 rpm on a magnetic stirrer (Cat#US152D, Bibby Scientific).

**TABLE 1:** Compositions and process parameters to prepare bimodal and unimodal constructs.

Scaffold type		Material properties				Process parameters				
		PCL (gr)	Acetic acid (ml)	Formic acid (ml)	Pyridine (ml)	Flow rate (ml/h)	Distance (cm)	Drum speed (RPM)	Needle diameter (G)	Voltage (kV)
Bimodal	8%	0.08	0.5	0.5	0.006	0.03	7	2000	21	9
	15%	0.15	0.5	0.5	0.02	0.06	7	2000	21	9
Unimodal	10%	0.10	0.5	0.5	0.006	0.03	7	2000	21	9

A co-electrospinning process (Figure 1B) was utilized to fabricate aligned nanofibers, which were then characterized. The concentrations of PCL were 8 percent and 15 percent, respectively. The solutions were poured into two syringes that were placed at a position facing one another and directed to the rotating drum collector. The voltage of the power supply was adjusted to 9 kV for both syringes, and the drum revolved at a speed of 2000 revolutions per minute. A 7 cm gap between the spinneret and the drum collector was established. On a stationary plate, unaligned fibers were created at 9 kV by feeding a 10 percent PCL solution at a flow rate of 0.03 ml/h (Figure 1C).

### 2.7 Mechanical properties of PCL constructs

The mechanical characteristics of PCL constructs were determined using uniaxial material testing equipment (MTS Criterion Model 43, MTS Systems Co., Eden Prairie, MN, United States) mounted with a one-kilogram load cell (Figures 1F,G). The specimens were cut in dimensions of 5 cm × 1 cm (length x width) and the thickness of the scaffold was measured using a digital caliper (Faithful Quality Tool, UK, Faicaldig digital caliper 150 mm) by placing the specimen between two glass slides. Tension was applied to PCL constructs (*n* = 5/group) using custom-made grips, and the constructs were strained with an initial 0.01N preloading. The test was performed until failure at a crosshead speed of 5 mm/min.

### 2.8 Scanning electron microscopy characterization

A turbo-pumped sputter coater (Quorum Q150T ES, UK) was used to coat the specimens with a 5 nm thickness of gold layer at a current of 20 mA. The constructs were examined at different magnifications using a Scanning Electron Microscope (JSM- IT200 (LA), JEOL, Japan). The diameters of scaffold fibers were measured using ImageJ (National Institute of Health, United States) software. The fiber diameter distribution of unaligned constructs and aligned constructs was measured using at least 150 fibers per picture (*n* = 5/group) following a similar method described in Section 2.5. In addition, the alignment of the PCL fibers was measured using ImageJ and reported in the form of frequency distribution.

### 2.9 Statistical analysis

The mechanical parameters of the aligned (bimodal) and unaligned (unimodal) constructs were compared with the mechanical properties of healthy rat ACL tissue using One-Way analysis of variance (ANOVA) with Tukey HSD (Honestly Significant Difference) post-hoc test. The fibril diameters and organizations of healthy and injured ACL tissues, the diameters of aligned and unaligned scaffold fibers as well as their organizations were compared using a t-test. The difference was considered significant when the *p*-value was less than 0.05 (*p* < 0.05).

## 3 Results

### 3.1 Diameter of collagen fibrils

**FIGURE 2:**
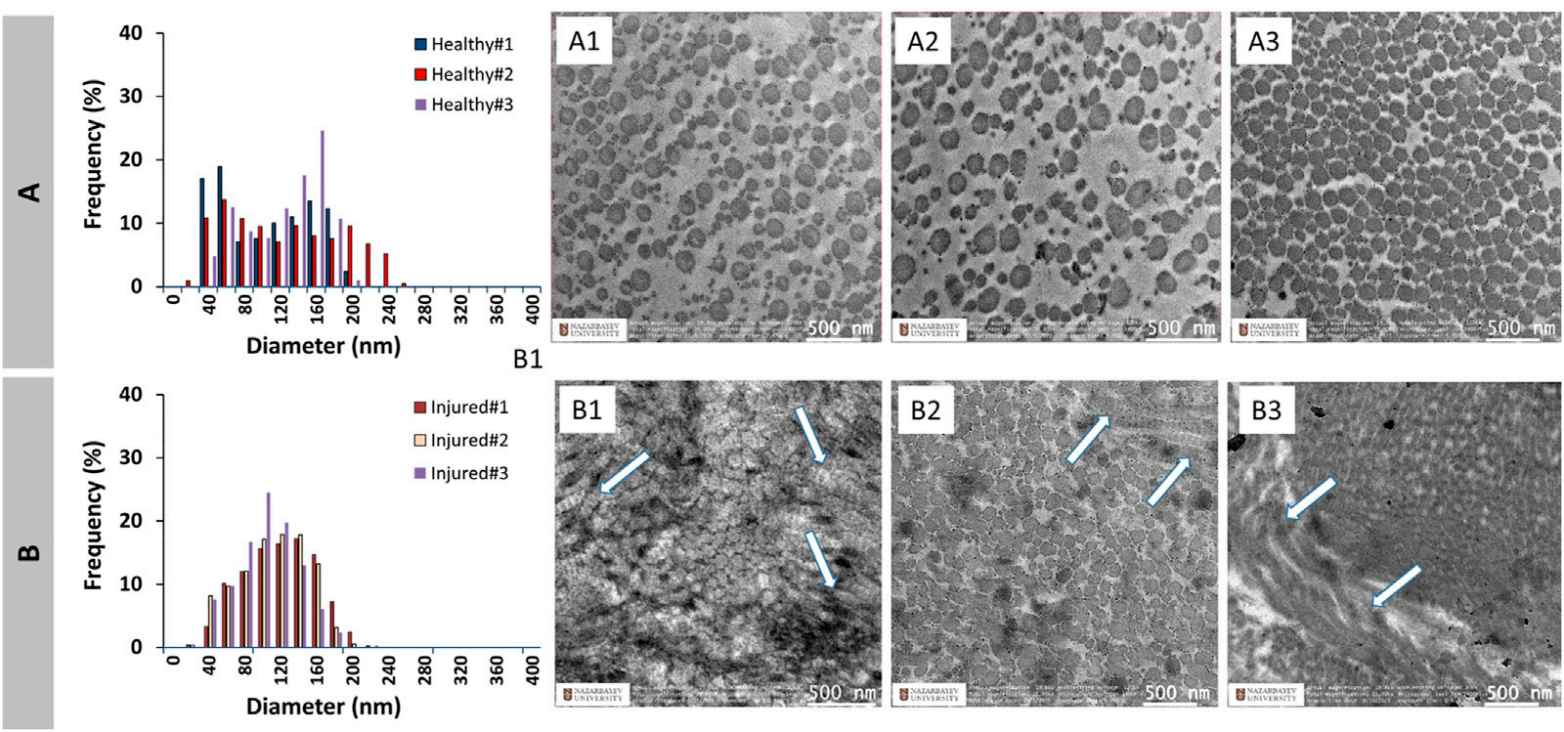
Diameter distribution of healthy **(A)** and injured **(B)** native ACL collagen fibrils and TEM images (Healthy: **A1–A3**, Injured: **B1–B3**). Arrows show disrupted alignment of some of the collagen fibrils upon injury.

The frequency distributions of collagen fibril diameter of healthy and injured ACL tissue are shown in Figures 2A,B, respectively. TEM photos of the corresponding histograms are also shown. The healthy ACL tissue appears to have a bimodal distribution, while the injured ACL tissue has a unimodal distribution. In addition, the healthy specimens showed a well-organized morphology, with collagen fibrils that were aligned in the longitudinal direction of the specimens (Figures 2A1–3) as determined by the roundness of the circles. However, TEM images of injured specimens demonstrate a disorganized structure as shown by fibrils aligned in diverse orientations on the specimens’ surfaces (Figures 2B1–3).

**FIGURE 3:**
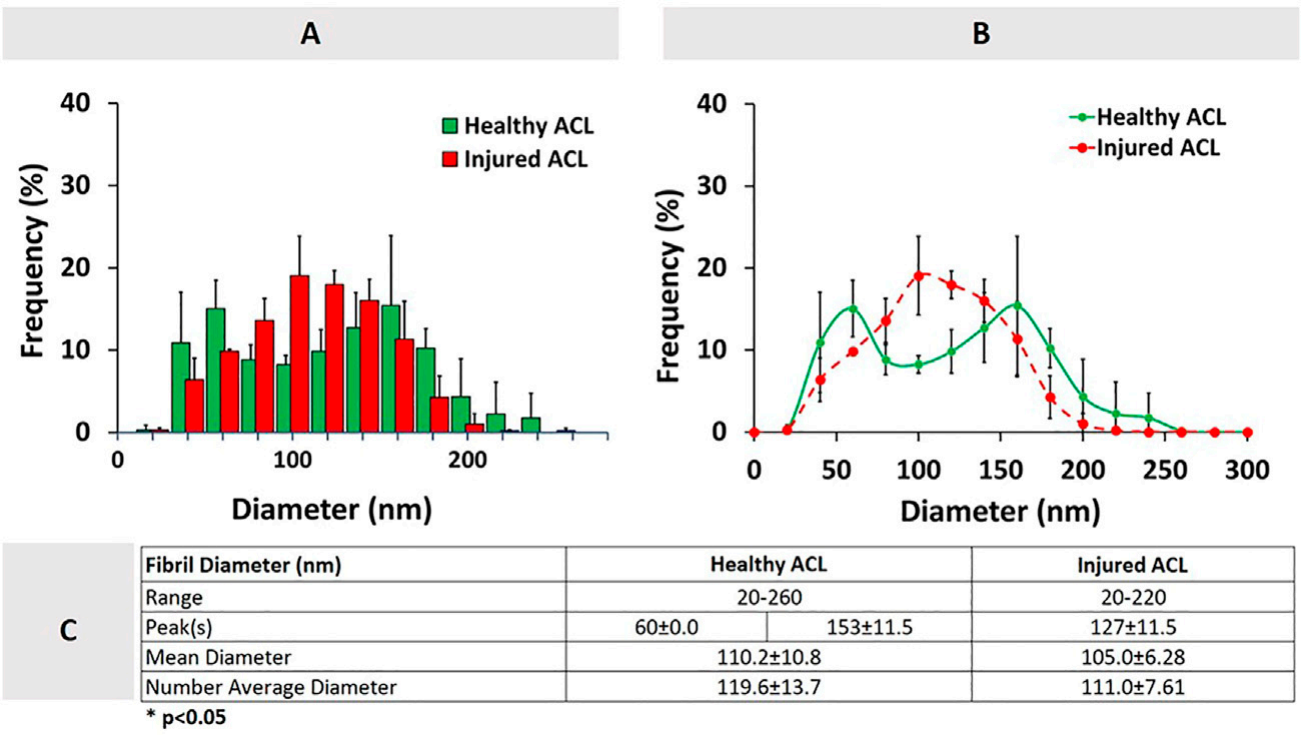
Healthy and injured ACL collagen fibril diameter distributions in the form of a histogram **(A)**, line graph **(B)**, and descriptive statistics **(C)**. Error bars show standard deviation (*n* = 3).

Figure 3 shows the combined distributions of ACL fibrils from injured and healthy ACLs. ACL tissue from the injured knee demonstrated a unimodal distribution with one peak at 127 ± 11.5 nm, while the healthy tissue showed a bimodal (two peaks) distribution with peaks at 60 ± 0.0nm and 153 ± 11.5 nm. A reduction in the diameter range of fibrils was observed from 20-260 nm to 20–220 nm, with the number average diameter value decreasing from 119.6 ± 13.7 nm to 111.0 ± 7.61 nm (Figure 3C). The mean diameter also decreased, going from 110.2 ± 10.8 nm to 105.0 ± 6.28 nm. Generally speaking, the diameter distribution of collagen fibrils in the rat ACL changed from a bimodal to a unimodal distribution after the injury, demonstrating a decrease in average diameters (*p* > 0.05).

### 3.2 Fiber diameter of PCL constructs

**FIGURE 4:**
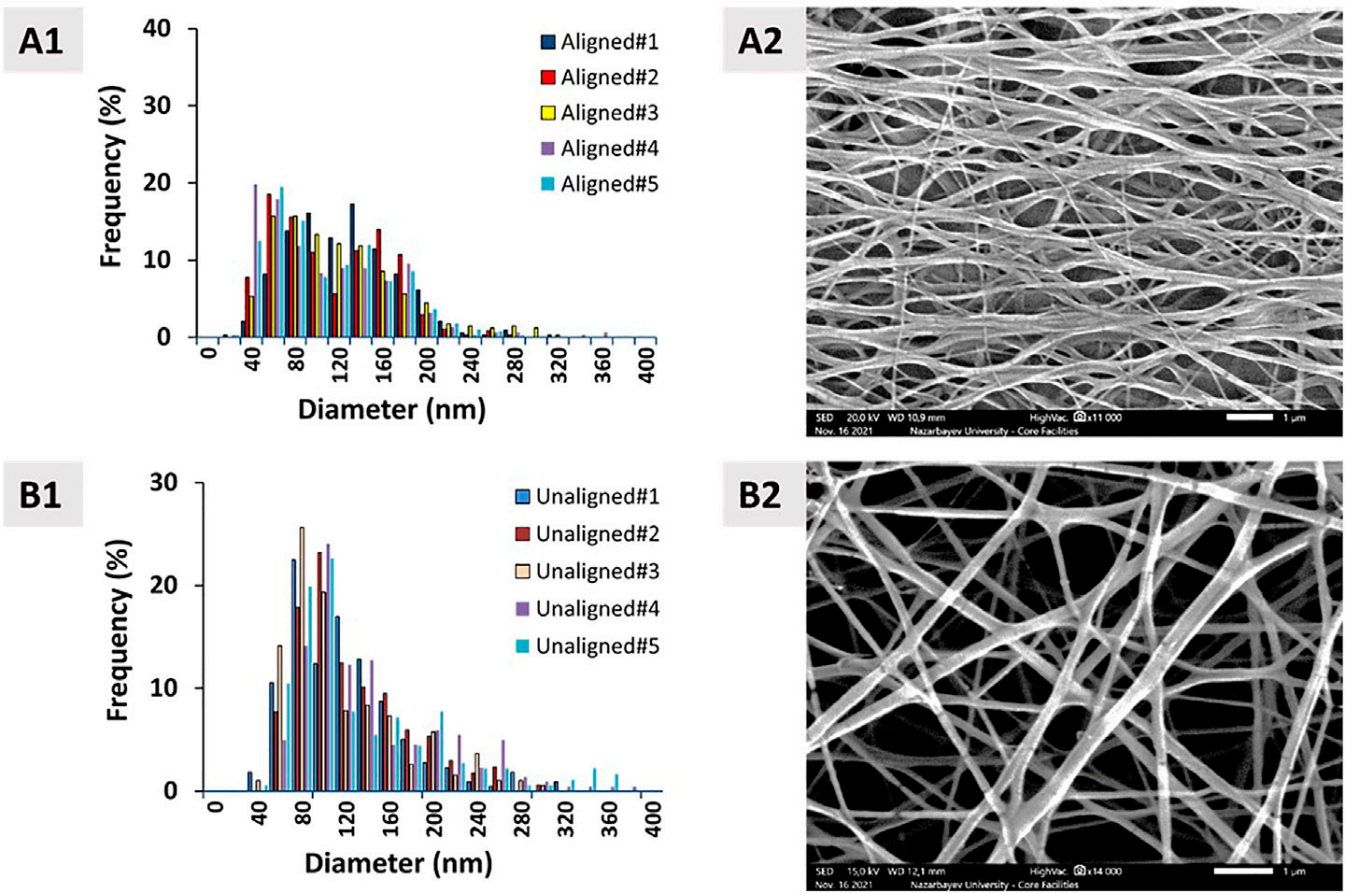
Diameter distribution of **(A1)** aligned and **(B1)** unaligned PCL fibers and corresponding representative SEM images (**(A2, B2)**, *n* = 5). Scale bar = 1 μm.

The histograms of the aligned (bimodal) and unaligned (unimodal) PCL constructs are shown in Figure 4, along with the corresponding SEM images of the constructs. The aligned constructs exhibited a bimodal diameter distribution (Figure 4A). The random constructs had a unimodal distribution (Figure 4B).

**FIGURE 5:**
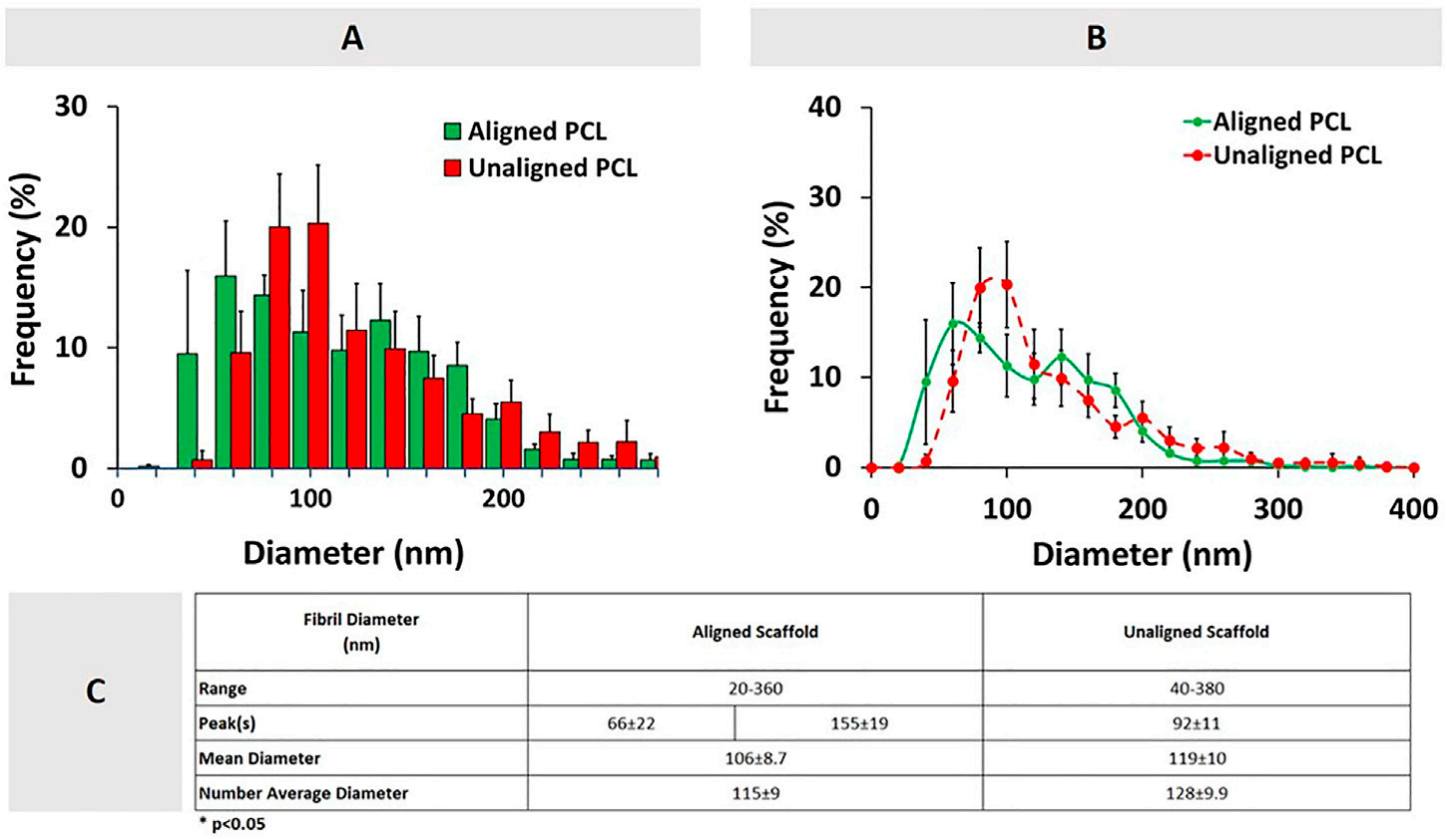
Combined aligned and unaligned PCL fiber diameter distributions in the form of **(A)** histogram, **(B)** line graph (*n* = 5), and **(C)** descriptive statistics. Error bars denote standard deviation.

Figure 5 demonstrates the combined fiber diameter distribution for PCL constructs that are aligned and unaligned. Results show that there is no statistically significant difference between the diameters of aligned and unaligned constructs in terms of mean diameter and number average diameter.

**FIGURE 6:**
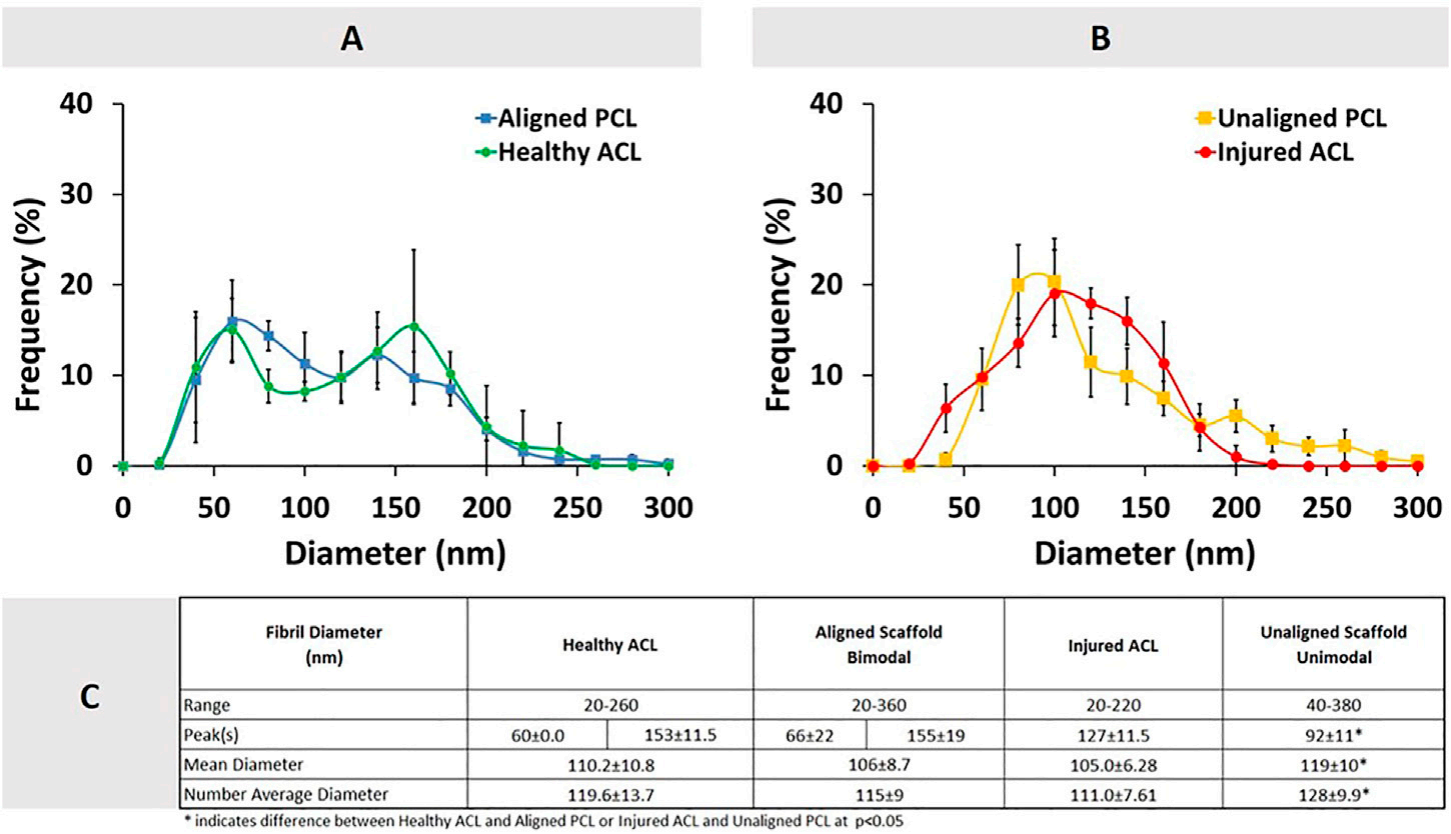
Comparison of diameter distributions between ACL tissue and PCL constructs. **(A)** Healthy ACL versus Aligned PCL, **(B)** Injured ACL versus Unaligned PCL, and **(C)** descriptive statistics. Error bars show standard deviation.

Comparison of ACL collagen fibril diameters and synthetic PCL construct fiber diameters are given in Figure 6. No statistically significant difference was observed between the diameters of aligned PCL fibers and healthy ACL fibrils (*p* > 0.05). Unaligned PCL scaffold fiber diameters, on the other hand, were found to be qualitatively similar yet quantitatively different from the injured ACL fibril diameters (*p* < 0.05).

**FIGURE 7:**
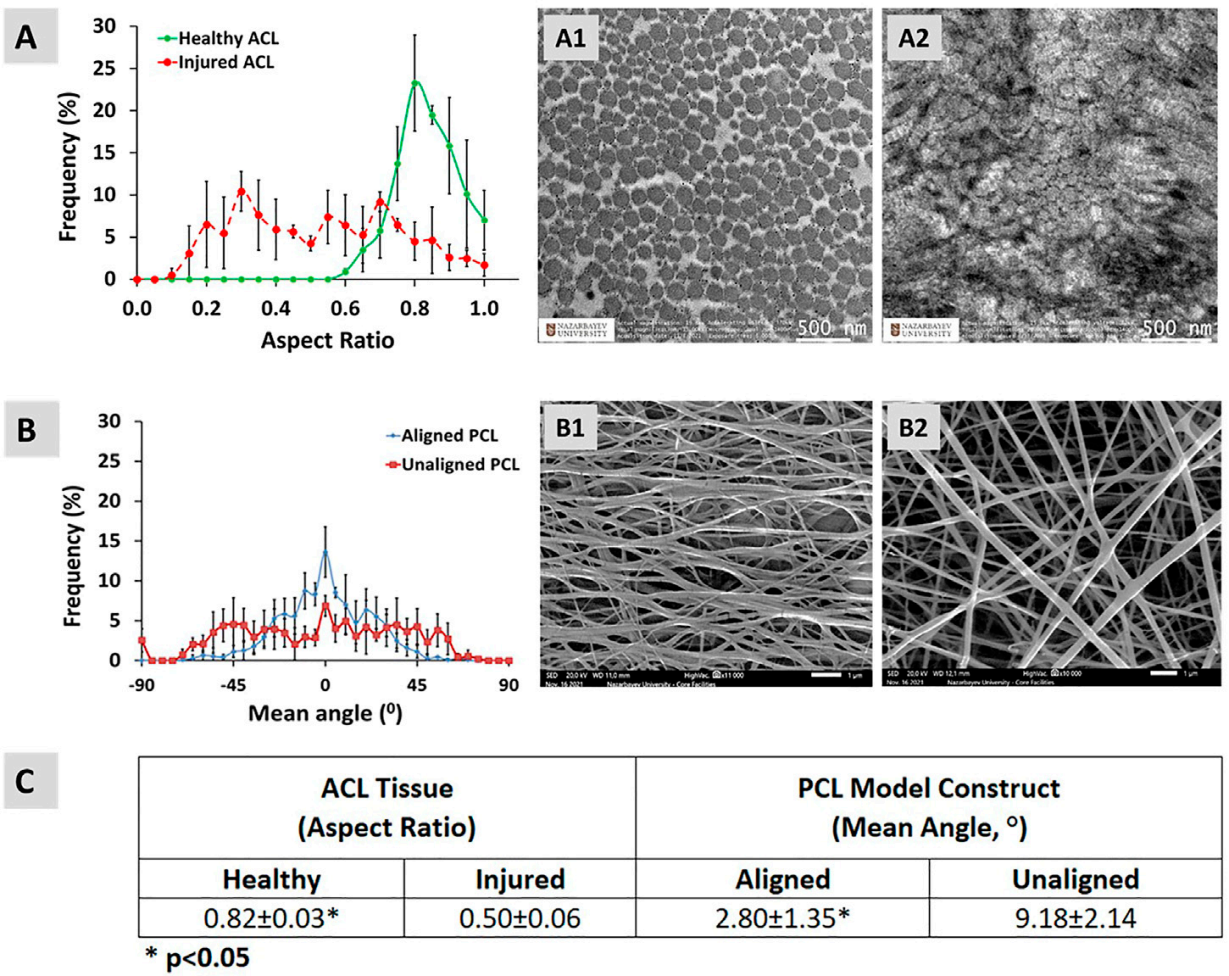
Alignment of **(A)** healthy and injured collagen fibrils in native ACL tissue (in the form of fibril cross-section aspect ratio), **(B)** PCL fibers, and **(C)** comparison between the pairs. Error bars represent standard deviation (*n* = 3). **(A1,A2)** are the representative TEM images for healthy and injured ACL tissue, respectively. **(B1,B2)** are the representative SEM images for aligned and unaligned PCL model constructs, respectively.

Fiber alignment of aligned and unaligned PCL constructs in the form of mean angle together with the alignment of collagen fibrils in healthy and injured ACL tissues in the form of aspect ratio are presented in Figure 7. The collagen fibrils in healthy ACL are seen round with an aligned organization (Figures 7A1,C), while the fibrils in injured ACL are disorganized (Figures 7A2,C). As clearly seen from Figure 7, the aligned bimodal constructs contained fibers aligned longitudinally as depicted by a normal mean angle distribution (Figures 7B1,C). Random unimodal constructs, on the other hand, depicted a flatter distribution of mean angle of fibers, indicating an obvious deviation from alignment (Figures 7B2,C).

### 3.3 Biomechanical characterization of ACL tissue and PCL constructs

**FIGURE 8:**
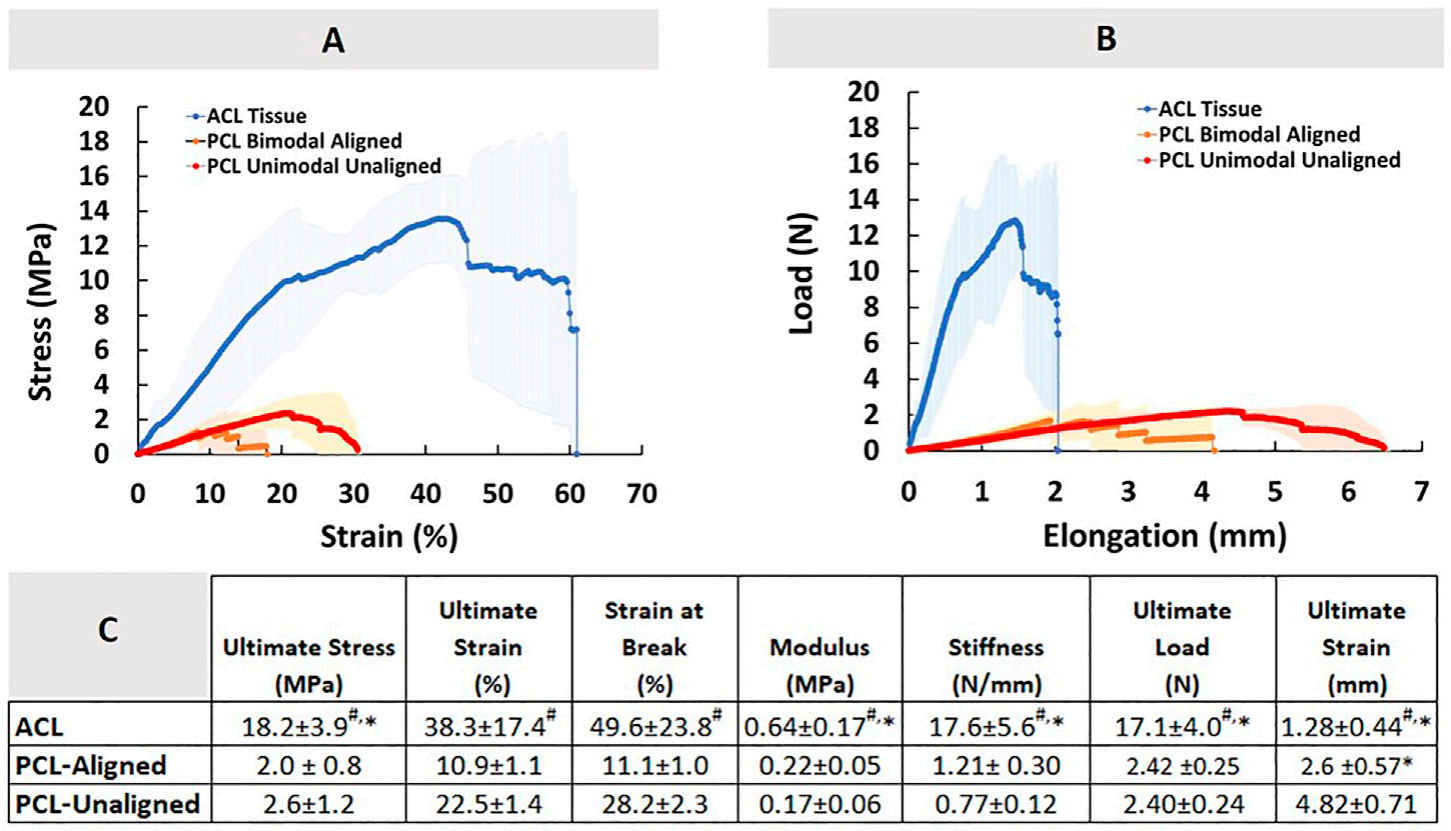
Comparison of Native ACL tissue and PCL constructs in terms of mechanical properties. **(A)** Stress-strain diagram, **(B)** Load-elongation diagram, and **(C)** descriptive statistics. An * indicates significant difference from PCL-Aligned, and ^#^ indicates significant difference from PCL-Unaligned at *p* < 0.05. Error bars represent SD.

The load-elongation and stress-strain behavior of healthy ACL tissue are shown in Figures 8A,B, and the values for each parameter tested are provided in Figure 8C. The ultimate stress and strain in the ACL tissue are 18.2 ± 3.9 MPa and 38.3 ± 17.4%, respectively. The tissue modulus was determined from the slope of the linear portion of the stress versus strain curve and is 0.64 ± 0.17 MPa. The stiffness was found to be 17.6 ± 5.6N/mm when the material was loaded. The ACL was stretched to an ultimate load of 17.1 ± 4.0N with an elongation of 1.28 ± 0.44 mm.

The load-elongation and stress-strain curves for the PCL constructs are also shown in Figures 8A,B. The tensile pattern of the PCL constructss was typical of tri-phasic tensile patterns, including toe, linear, and yield regions. The results for the typical mechanical performance parameters of PCL constructs are also shown in Figure 8C. The ultimate stress and strain of the aligned PCL model constructs were determined to be 2.0 ± 0.8 MPa and 10.9 ± 1.1%, respectively, in the aligned PCL constructs. Similarly, the ultimate stress and strain of the unaligned PCL constructs, which represented the injured ACL tissue, were 2.6 ± 1.2 MPa and 22.5 ± 1.4%, respectively. The moduli of the constructs with the aligned and unaligned organization were 0.22 ± 0.05 MPa and 0.17 ± 0.06 MPa, respectively. The stiffness of aligned and unaligned constructs was found to be 1.21 ± 0.30N/mm and 0.77 ± 0.12N/mm, respectively, for the two categories of constructs. Considering aligned and unaligned constructs, the ultimate tensile load was 2.42 ± 0.25 N and 2.40 ± 0.24 N, respectively, when they are stretched to an ultimate elongation of 2.6 ± 0.57 mm and 4.82 ± 0.71 mm, respectively.

Comparison of aligned and unaligned PCL constructs revealed a significant difference only in the ultimate strain measured in true unit (mm), all other being similar. However, PCL constructs regardless of the type of fiber organization were found mechanically inferior to the native tissue (*p* < 0.05).

## 4 Discussion

The distributions of collagen fibril diameter in rat ACL tissue before and after injury, as well as the tensile properties of healthy ACL tissue were investigated in this study, which is solely on the design, fabrication and characterization of a biomaterial model. To establish the appropriateness of the PCL constructs for ACL repair/regeneration, the tensile properties as well as their fiber diameter distributions were also investigated. There are certainly options for the materials of construction of our nanofiber-based model construct, including poly (lactic-co-glycolic acid), PLGA, and poly (L-lacticacid), PLLA. Our previous investigations on PLGA nanofiber scaffolds for the *in-vitro* activity of human tendon fibroblasts (Erisken et al., 2013) demonstrated that PLGA shrinks when it comes into contact with cell culture media. An ACL model construct made of PLGA would get shorter in length in an *in-vivo* test, and restrict the mobility of the knee joint relative to the native ACL. PLLA is indeed a more relevant material in terms of biomechanical properties and cell attachment. However, it is a bulk eroding biomaterial. Fibers made of PLLA could weaken mechanically in an accelerated manner upon progression of erosion, leaving the graft mechanically inferior when implanted. In this study, we preferred PCL, despite its initially weaker mechanical properties, because degradation would progress from the surface, making the fibers thinner with time. However, this thinning is expected to be compensated by the ECM components deposited around the fibers by the cells. The initially weaker mechanical properties will eventually be improved. Regarding attachment of cells, PCL constructs are relatively hydrophobic. However, our earlier studies involving electrospun PCL scaffolds seeded with MC3T3 and adipose derived stromal cells (Erisken et al., 2008b; Erisken et al., 2011) demonstrate that the cells do like the surface of PCL nanofibers, attach and perform their activities. A combination of PLLA and PCL can optimize the construct properties, and this could be an option for our planned biocompatibility test for the proposed model construct.

The rat ACL’s collagen fibril diameter distribution shifted from bimodal to unimodal after rupture, with a reduction in the mean diameter of the collagen fibrils. The fiber diameter distributions of electrospun PCL constructs were designed to have a bimodal and unimodal mode, enabling it to look qualitatively and quantitatively similar to healthy and injured rat ACL tissues. ACL collagen fibers are structured into fibril bundles that produce an orderly parallel wave pattern along the long axis of the collagen fibers, as seen in the TEM images. The mechanical properties of ACL are enhanced as a result of this hierarchical configuration (Muellner et al., 2001). Our findings demonstrated that the ACL tissue of a healthy rat has a well-organized/aligned pattern of collagen fibrils, which is altered when the tissue is injured. Similar to what is observed in the rat ACL tissue, a ruptured bovine (Beisbayeva et al., 2021), sheep (Smatov, 2021) and human (Hashemi et al., 2008) ACL was found to have a disorganized collagen fibril pattern.

Previous studies on the bovine ACL revealed diameter peaks of around 75nm and 210 nm before the injury, which then changed to a single peak of 100 nm after injury (Beisbayeva et al., 2021). A similar alteration occurred in the collagen fibril diameter of human ACL, which had an average diameter of 75 nm (20–185 nm range) before decreasing to 71 nm (20–290 nm range) after injury. Also, previous studies on the sheep ACL showed diameter peaks of around 75 nm and 160 nm before the injury, which then changed to a single peak of 101 nm after injury (Smatov, 2021). The diameter distribution of collagen fibrils in the ACL of healthy and injured rats was investigated in this study. The results revealed that the diameter of fibrils reduced (based on average, *p* > 0.05) following mechanical deformation and this resulted in a change in the modality from bimodal to unimodal. Both the findings obtained by Beisbayeva et al. (2021) on bovine ACL tissue, Smatov (2021) on sheep ACL tissue and our results obtained on rat ACL tissue demonstrate similar behavior in terms of the distribution of diameters. In these investigations, the mean peak fibril diameters (small/large peak diameters) of healthy bovine and sheep ACL tissue were 73.3 ± 11.5 nm/213 ± 11.5 nm and 75.6 ± 8.5 nm/157.6 ± 3.8 nm, respectively. The mean peak fibril diameters of rat ACL tissue were 60 ± 0.0 nm/153 ± 11.5 nm. Despite differences in the mean peak values (no statistical analysis), all three distributions qualitatively look similar.

The electrospinning technique was used to create PCL electrospun constructs that represented the healthy and injured states of rat ACL tissues. The distribution of collagen fibril diameter of healthy and injured ACLs was represented by the aligned and unaligned PCL constructs, respectively. The ACL collagen fibrils in healthy state was seen to be both quantitatively and qualitatively imitated by PCL constructs with aligned structures. Unaligned constructs were unable to mimic the distribution of injured ACL tissue quantitatively despite the observed similarity in frequency distributions. Therefore, a fine tuning is needed in the composition of PCL solution to fabricate unimodal constructs. Even though the electrospinning approach is an excellent technique for the production of nanoscale constructs, producing fibers with controlled diameter requires tight control over the processing and material parameters (Erisken et al., 2013).

There has been evidence that changes in the mean diameter and distribution of collagen fibrils in the ACL are important markers of tissue mechanics, and it has been shown that variations in fibril diameter, as well as distribution, have a direct influence on mechanical characteristics (Marieswaran et al., 2018). Bimodal distribution, such as seen in healthy ACL tissue, results in greater mechanical properties because the interfibrillar gaps between larger fibrils are filled with thinner fibrils to generate a densely packed ECM. This hierarchical structure of ligament tissue is altered upon deformation, reducing the ligament tissue’s capacity to withstand physiological stresses, and making the tissue mechanically more sensitive to injury or rupture. It should be highlighted that the mechanical parameters of healthy ACL tissue were the only ones investigated in this research. Due to unavailability of tools to measure the mechanical properties of the injured ACL tissue, it has not been possible to compare the mechanical properties of injured ACL with the healthy ACL and the PCL constructs.

Each of the parameters examined in this research, including ultimate strain, ultimate stress, stiffness, and modulus, showed that the native ACL tissue outperformed both aligned and unaligned PCL constructs. A comparison of the two groups of constructs showed that the aligned and unaligned PCL constructs were similar in terms of all the parameters tested except ultimate strain.

## 5 Conclusion

This study investigated the morphological and mechanical features of the native rat ACL tissue. The collagen fibril diameter distribution shifted from bimodal to unimodal when the rat ACL ruptured, with a subsequent decrease in the average fibril diameter. The fiber diameter distributions of bimodal PCL constructs both quantitatively and qualitatively matched the collagen fibril diameter distribution of healthy ACL tissue, accompanied with significantly lower mechanical properties. To the best of our knowledge, there is currently no published research on the utilization of nanofiber-based model constructs with bimodal distribution for ACL reconstruction/regeneration. The bimodal and unimodal synthetic constructs that are designed and fabricated here could be utilized to evaluate the behavior of ligament fibroblasts to understand the mechanism of tissue healing to be later used as a synthetic construct in ACL reconstruction. This is expected to have a significant impact on the improvement of orthopedic related research.

## Data Availability

The raw data supporting the conclusion of this article will be made available by the authors, without undue reservation.
